# Cell adhesion molecules as potential biomarkers of nephritis, damage and accelerated atherosclerosis in patients with SLE

**DOI:** 10.1177/0961203314528061

**Published:** 2014-07

**Authors:** S Skeoch, S Haque, P Pemberton, IN Bruce

**Affiliations:** 1 Arthritis Research UK Centre for Epidemiology, Centre for Musculoskeletal Research, Institute for Inflammation and Repair, University of Manchester, Manchester Academic Health Sciences Centre, Manchester, UK; 2 Department of Rheumatology, University Hospital of South Manchester, Manchester, UK; 3 Specialist Assay Laboratory, Central Manchester University Hospitals NHS Foundation Trust, Manchester, UK; 4 The Kellgren Centre for Rheumatology, NIHR Manchester Musculoskeletal Biomedical Research Unit, Central Manchester University Hospitals NHS Foundation Trust, Manchester Academic Health Science Centre, Manchester, UK

**Keywords:** Systemic lupus erythematosus, cardiovascular disease, nephritis, cellular adhesion molecules

## Abstract

**Objectives:**

The aim of the current study was to compare levels of vascular cell adhesion molecule-1 (VCAM-1) and E-selectin in lupus patients and controls and to investigate their association with clinical phenotype, disease activity and damage.

**Methods:**

We compared levels of serum VCAM-1 and E-selectin in 178 female lupus patients and 69 age-and sex-matched controls. Using linear regression we also examined the association between these markers and disease activity, damage, renal and skin involvement as well as clinical and subclinical cardiovascular disease.

**Results:**

E-selectin was increased in patients compared to controls (median (IQR) 10.5 (6.85, 13.9) vs 7.86 (5.39, 10.4) ng/ml; *p* < 0.001). E-selectin was also associated with overall damage and carotid plaque (β (95% confidence interval): 0.27 (0.029, 0.511) and 0.26 (0.148, 0.507)). Whilst there was no significant difference in VCAM-1 levels between groups overall, we found a significant association between VCAM-1 and with active renal disease (β (95% confidence interval): 1.10 (0.69, 1.51)).

**Conclusions:**

E-selectin may act as a marker of cardiovascular risk in SLE, whilst VCAM-1 may have a role as a non-invasive biomarker for lupus nephritis activity.

## Background

The cellular adhesion molecules (CAMs), vascular cell adhesion molecule-1 (VCAM-1) and E-selectin, are expressed exclusively on the surface of endothelial cells. Their function is to facilitate leucocyte-endothelial cell interactions and the transmigration of inflammatory cells to sites of inflammation. Their expression is upregulated by pro-inflammatory cytokines such as tumour necrosis factor (TNF)-alpha. These molecules are also shed into the circulation and may act as markers of endothelial activation and dysfunction.

VCAM-1 and E-selectin have been found to be elevated in patients with systemic lupus erythematosus (SLE).^1,2^ Some, but not all studies, have also found correlations with disease activity and particular disease phenotypes.^3,4^ For example, VCAM-1 appears to be of particular interest in renal disease, as raised levels have been found in active nephritis.^5,6^ In contrast, E-selectin levels have been less consistently associated with disease activity. However, one study did find increased E-selectin levels in patients with cutaneous manifestations.^7^

In the general population, levels of CAMs have also been found to predict cardiovascular events and have been associated with hypertension.^8^ In SLE, where cardiovascular disease (CVD) is a leading cause of death,^9^ Gustafsson et al. found that high circulating levels of VCAM-1 were associated with cardiovascular mortality over a 12-year follow-up in an SLE cohort. Other studies have found associations with E-selectin and markers of subclinical CVD but results have been inconsistent. Such variation in results may reflect small sample sizes and heterogeneity of populations studied.

The aim of this study was to compare levels of VCAM-1 and E-selectin in a large cohort of lupus patients and a control population. We also investigated their association with lupus phenotype, in particular renal disease, skin disease and CVD as well as overall SLE disease activity and damage.

## Methods

### Study design and recruitment

We carried out a cross-sectional study of adult female SLE patients and age- and sex-matched controls between August 2007 and July 2009. Ethical approval was obtained from the North West Regional Ethics Committee and informed consent was given by all participants. Patients were recruited from outpatient clinics in the North West of England. Controls were recruited via a “best friend” system, whereby patients invite a friend to participate in the study. All patients met four or more of the 1997 American College of Rheumatology (ACR) revised classification criteria for SLE.^10^ Exclusion criteria included pregnancy and/or lactation within the past three months, recent infection within the past month and malignancy within the last 12 months.

### Data collection

All subjects underwent a clinical assessment including history of demographics, lifestyle factors and medical history, and where necessary a review of the medical notes was carried out. The clinical phenotype and distribution of SLE manifestations were recorded. Disease activity was assessed using the Systemic Lupus Erythematosus Disease Activity Index (SLEDAI)-2000 (SLEDAI-2K) score.^11^ Active muco-cutaneous disease and active nephritis were defined as being present in patients scoring on either organ system within the relevant SLEDAI-2 K domains. A diagnosis of lupus nephritis ‘ever’ was recorded in patients with biopsy-proven disease or in patients with a history of haematuria and/or proteinuria and/or renal impairment who had a clinical diagnosis of nephritis in the absence of a formal biopsy. Damage was scored using the Systemic Lupus International Collaborating Clinics (SLICC) Damage Index (SDI).^12^ Fasting blood samples were drawn and, after centrifugation (1500 g for 15 minutes), plasma was stored at –80℃. Levels of E-selectin and VCAM-1 were measured using Duoset enzyme-linked immunosorbent assay (ELISA) development assays from R&D Systems (Abingdon, UK). The E-selectin assay had a working range up to 6 ng/ml with a minimum detection limit of 0.1 ng/ml. The intra-assay coefficient of variation (CV) was 6% and the inter-assay CV was 7%. The VCAM assay had a working range up to 1 ng/ml with a minimum detection limit of 0.04 ng/ml. The intra-assay CV was 3% and the inter-assay CV was 8%.

Carotid ultrasound was performed, specifically evaluating carotid intima-medial thickness (IMT) and presence of carotid plaque. Measurements were taken using previously validated methodology.^13^ In brief, three sets of IMT measurements were taken 1  cm from the carotid bulb bilaterally. From these, a mean IMT was calculated. Carotid plaque was defined as presence of two or more of the following parameters: IMT >1.5 mm, wall protrusion of >50% or increased wall echogenicity.

### Statistical analysis

Statistical analysis was performed using STATA11 software (StataCorp LP, TX, USA). Non-parametric tests (Mann-Whitney *U* test and Kruskal-Wallis test) were used to compare variables in patients and control subjects. VCAM-1 and E-selectin levels were log-transformed to satisfy the assumption of normality for linear regression. Linear regression models were employed to evaluate associations of levels of VCAM-1 and E-selectin with specific lupus features, SLEDAI-2K, SDI, and the presence of clinical and subclinical CVD. Significance level was set at *p* < 0.05. Our multivariable models included variables with a *p* value <0.2 on univariate analysis.

## Results

### Patient characteristics

We studied 178 patients and 69 controls with a median (interquartile range (IQR)) age of 53 (46, 61) and 50 (39, 60) years old, respectively (*p* = 0.066) (Table 1). Overall 26 (14.7%) patients and one (1.5%) control had a history of CVD (*p* = 0.004).

**Table 1:** Cohort description: All results are displayed as median (IQR) or as *n* (%)^a^ where indicated

Characteristic	Patients (*n* = 178) Median (IQR)/frequency(%)	Controls (*n* = 69) Median (IQR)/ frequency(%)	*p* value
Age	53 (46, 61)	50 (46, 61)	0.066
Smokers^a^	16 (8.9)	9 (13.2)	0.36
Clinical CVD^a^	26 (14.8)	1 (1.5)	0.004
Current statin therapy^a^	48 (27.8)	4 (5.79)	<0.01
Current anti-hypertensive therapy^a^	69 (38.76)	7 (10.29)	<0.01
Systolic blood pressure (mmHg)	127.5 (115, 143)	120.5 (110, 135)	0.030
Diastolic blood pressure (mmHg)	71 (64, 76)	68 (60, 74)	0.026
Disease duration (years)	13 (7, 23)	–	–
SLEDAI score	2 (0, 4)	–	–
Active disease (SLEDAI>3)^a^	53 (30)	–	–
Evidence of SLE-related damage (SLICC-DI>0)^a^	110 (61.8)	–	–
SDI	1(0, 2)	–	–
Active nephritis^a^	5 (2.8)	–	–
Inactive nephritis^a^	31 (17.4)	–	–
Active muco-cutaneous disease^a^	52 (32.1)	–	–
Carotid plaque^a^	78 (43.8)	–	–
Intimal medial thickness (mm)	0.0633 (0.0533, 0.0733)	–	–
VCAM-1 (ng/ml)	274 (226.1, 351.9)	268.5 (226, 372)	0.95
E-selectin (ng/ml)	10.5 (6.85,13.9)	7.86 (5.39, 10.4)	<0.001

IQR: interquartile range; CVD: cardiovascular disease; SLEDAI: Systemic Lupus Erythematosus Disease Activity Index; SLE: systemic lupus erythematosus; SLICC-DI: Systemic Lupus International Collaborating Clinics Damage Index; VCAM: vascular cell adhesion molecule-1.

The cohort included 160 (91%) Caucasians. The median (IQR) disease duration and SLEDAI-2K scores were 13 (7, 23) years, and 2 (0, 4) respectively. In total, 53 (30%) patients had a SLEDAI-2K >3. The median (IQR) SDI was 1 (0, 2) with 110 (61.8%) having at least one item of damage in the SDI. Five patients had active nephritis as defined using the SLEDAI-2 K score. All five patients had biopsy-proven lupus nephritis (class III-V) (see Appendix, Table A1).

Carotid plaque was observed in 78 (43.8%) patients on ultrasound and the median (IQR) IMT for the population was 0.063 (0.053, 0.073) cm. A greater number of SLE patients were on anti-hypertensive agents and statins than the control population (39% vs 10%, *p* < 0.01 and 28 vs 6%, *p* = 0.026, respectively).

#### CAMs in patients and controls

The median (IQR) E-selectin levels were significantly higher in patients compared to controls (10.5 (6.9, 13.9) vs 7.9 (5.4, 10.4) ng/ml, respectively; *p* < 0.001). VCAM-1 levels did not significantly differ between groups (Table 1).

#### VCAM-1 in SLE patients

The distribution of VCAM-1 was skewed and was log-transformed for analysis. VCAM-1 was significantly associated with age (β coefficient (95% confidence interval (CI)): 0.006 (0.001, 0.011)), therefore analysis was age-adjusted. The median (IQR) VCAM-1 levels were also significantly higher in patients with active nephritis than those with inactive nephritis and those with no history of nephritis (515.5 (307.6, 929) vs 276.7 (199.2, 351.9) vs 272.3 (223.6, 343.5) ng/ml, respectively; *p* < 0.001). In an age-adjusted linear regression model, VCAM-1 was significantly associated with active nephritis and inversely associated with smoking (Table 2). These associations remained in a multivariable model (β coeff (95% CI): 1.10 (0.69, 1.51) and −0.247 (−0.435, −0.058) respectively).

**Table 2:** Associations of VCAM-1^a^ with SLE characteristics

Characteristic	Age-adjusted β coefficient (95% CI)	*p* value	Fully adjusted β coefficient (95% CI)	*p* value
Age	–	0.005 (−0.001, 0.010)	0.52
Disease duration (years)	0.005 (−0.001, 0.104)	0.75	–
Active disease (SLEDAI>3) (%)	−0.096 (−0.217, 0.167)	0.121	−0.076 (−0.194,0.041)	0.199
Evidence of SLE-related damage (SLICC>0) (%)	0.047 (−0.049, 0.167)	0.392
Active nephritis	0.658 (0.035, 0.161)	<0.001	1.10 (0.69, 1.51)	**<**0.01
Inactive nephritis	0.019 (−0.122, 0.161)	0.784	–
Active muco-cutaneous disease	−0.70 (−0.193, 0.053)	0.26	–
Clinical CVD	−0.78 (−0.231, 0.0748)	0.315	–
Carotid plaque presence	0.37 (−0.088, 0.163)	0.56	–
Intimal medial thickness	0.146 (−3.076, 2.67)	0.396
Current smoking	−0.261 (0.442, −0.8028)	0.005	−0.247 (−0.435, −0.058)	0.01
Current anti-hypertensive therapy	−0.097 (−0.21, 0.015)	0.09	−0.09 (−0.20, 0.03)	0.146
Systolic blood pressure	1.1 × 10^−5^ (−0.002, 0.002)	0.966	–	–
Diastolic blood pressure	−0.001 (−0.007, 0.004)	0.676	–	–

a VCAM-1 levels were log transformed for this analysis. VCAM-1: vascular cell adhesion molecule-1; SLEDAI: Systemic Lupus Erythematosus Disease Activity Index; SLE: systemic lupus erythematosus; CVD: cardiovascular disease; SLICC-DI: Systemic Lupus International Collaborating Clinics Damage Index; CI: confidence intervals.

#### E-selectin in SLE patients

In an age-adjusted linear regression model, E-selectin was associated with carotid plaque and the SDI but not with overall disease activity or other SLE features (Table 3). In a multivariable model, both carotid plaque and SLE-related damage remained independently associated with levels of E-selectin (β coeff (95% CI): 0.261 (0.015, 0.507) and 0.271 (0.029, 0.511), respectively).

**Table 3:** Associations of E-selectin^a^ with SLE characteristics

Characteristic	Age-adjusted β coefficient (95% CI)	*p* value	Fully adjusted β coefficient (95% CI)	*p* value
Age	–	–	−0.003 (−0.015, 0.081)	0.576
Disease duration (years)	−0.0002 (0.007, 0.011)	0.61	–	–
Active disease (SLEDAI>3) (%)	0.129 (−0.082, 0.341)	0.23	–	–
Evidence of SLE-related damage (SLICC >0) (%)	0.236 (0.044, 0.427)	0.02	0.270 (0.029, 0.511)	0.028
Active nephritis	0.082 (−0.476, 0.640)	0.77	–	–
Inactive nephritis	0.048 (−0.203,0.300)	0.71	–	–
Active muco-cutaneous disease	0.054 (−0.160, 0.053)	0.617	–	–
Clinical CVD	−0.098 (−0.362, 0.165)	0.46	–	–
Carotid plaque presence	0.268 (0.0478, 0.488)	0.017	0.261 (0.0149, 0.507)	0.038
Intimal medial thickness	−0.327 (−5.432,4.779)	0.9	–	–
Current smoking	0.193 (−0.122,0.510)	0.23	–	–
Current anti-hypertensive therapy	0.069 (−0.124, 0.26)	0.48	–	–
Systolic blood pressure	−0.002 (−0.006, 0.003)	0.418	–	–
Diastolic blood pressure	−0.001 (−0.0104, 0.007)	0.78	–	–

a E-selectin levels were log transformed for this analysis. SLEDAI: Systemic Lupus Erythematosus Disease Activity Index; SLE: systemic lupus erythematosus; CVD: cardiovascular disease; SLICC-DI: Systemic Lupus International Collaborating Clinics Damage Index; CI: confidence intervals.

## Discussion

In this large cross-sectional cohort, we found raised circulating levels of E-selectin in SLE patients compared with controls and that E-selectin was independently associated both with SLE-related damage and also with carotid plaque. A number of previous studies have also reported raised E-selectin in SLE, but interestingly, few have found a correlation with disease activity.^4^ Two small longitudinal studies found that levels of E-selectin varied little on repeated measurement even in inactive disease, suggesting that low-grade endothelial activation may persist even in the absence of significant clinical disease activity.^3,4^ Chronic endothelial activation may contribute to the accelerated atherosclerosis seen in SLE. This could explain the association of E-selectin levels with markers of subclinical atherosclerosis found in this and other studies.^14,15^ Rho et al. found that E-selectin and VCAM-1 were significantly associated with coronary artery calcification, independent of other Framingham risk factors while Reynolds et al. recently described an association with persistently raised E-selectin levels and carotid plaque. E-selectin therefore could act as a novel risk marker for atherosclerosis in SLE; however, larger longitudinal studies would be required to investigate this further. The association of E-selectin with SLE-related damaged had been less well studied; however, Rho et al. also found an association with SDI and E-selectin.^15^ Patients with SLE-related damage are likely to have had more severe or poorly controlled disease, which may lead to chronic endothelial activation as reflected by raised E-selectin levels.

**Figure 1:**
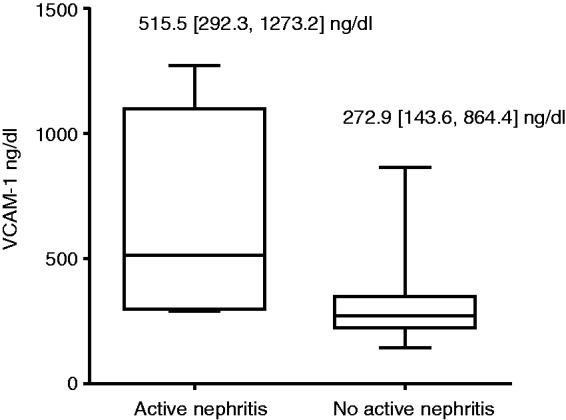
Levels of vascular cell adhesion molecule-1 (VCAM-1) in patients with and without active nephritis (median (maximum, minimum)).

Whilst we found no significant difference in VCAM-1 levels in patients and controls, nor an association with disease activity, there was a marked association with active lupus nephritis. Although only five patients had active nephritis, the significant increase in levels was not driven by a single outlier and we observed a genuine trend for higher VCAM-1 levels in all cases with active nephritis (see Figure 1). This association has also been noted in a number of other studies. For example, Singh et al. described a close correlation between urinary VCAM-1 and nephritis activity on biopsy.^6^ In other small studies VCAM-1 levels rose prior to a flare and persistently raised levels following clinical resolution was associated with subsequent relapse.^3,16^ The current gold standard for monitoring renal disease activity is serial renal biopsy; however, VCAM-1 could provide an alternative non-invasive monitoring tool. We did not find an association with VCAM-1 and overall disease activity using the SLEDAI-2K. We recognise that two-thirds of patients had low disease activity at the time of assessment and therefore our study may lack the power to detect an association between non-renal inflammation and VCAM-1. Our data do, however, suggest that the association seems specific for active renal disease.

The negative association with smoking and VCAM-1 was surprising. Smoking is associated with immune activation and it would be expected that smokers may have higher levels of VCAM-1. On review of the literature, however, few studies have found an association with serum VCAM-1 and smoking; in fact, one study found an inverse association in lupus patients.^17^ In vitro studies have shown some increased expression of VCAM-1 on the surface of endothelial cells following exposure to nicotine.^18^ The relationship of serum VCAM-1 and smoking status is not yet clear and there may be unmeasured confounding contributing to our result.

Although this is one of the largest studies evaluating cellular adhesion molecules in SLE patients, the study was not powered to detect associations with clinical CVD. Additionally, the current study population was predominantly Caucasian, hence these results may not be generalisable to other ethnicities.

Carotid plaque burden was not measured in the control cohort. As plaque prevalence is significantly lower in the general population, a much larger sample size would have been required to test associations with plaque in the control group and this was not within the scope of this study. As this is a cross-sectional study, we cannot drawn any conclusions about the temporal relationship of CAMs and SLE disease characteristics. Any biomarker should not only be associated with the biological process of interest but must be sensitive to change, thus longitudinal studies are planned to validate the use of these molecules as biomarkers in SLE.

Despite these limitations, both E-selectin and VCAM-1 could have utility in clinical practice. E-selectin may help identify patients at increased risk of overall damage and specifically of CVD. VCAM-1 may be a useful biomarker for renal disease activity and supplement current indirect markers of lupus nephritis.

## Appendix

**Appendix Table A1:** Characteristics of active nephritis patients

Patient 1	Class III nephritis, haematuria, proteinuria^a^ (1.2 gm/24 hours), hypocomplementaemia, eGFR = 59 ml/min/1.73 m^2^
Patient 2	Class IV nephritis, proteinuria^a^ (1.5 gm/24 hours), elevated anti-dsDNA antibodies, hypocomplementaemia, eGFR = 51 ml/min/1.73 m^2^
Patient 3	Class V nephritis, proteinuria (albumin:creatinine ratio:144 mg/mmol)^a^, hypocomplementaemia, elevated ds-DNA, eGFR = 52 ml/min/1.73 m^2^
Patient 4	Class V nephritis, proteinuria (on dipstick), pyuria (sterile)^c^, elevated anti-dsDNA antibodies, eGFR = 49 ml/min/1.73 m^2^
Patient 5	Class III nephritis, haematuria^b^, proteinuria (albumin:creatinine ratio 44)^a^ hypocomplementaemia, eGFR = 68 ml/min/1.73 m^2^

a Proteinuria present as per SLEDAI-2 K definition. ^b^Haematuria present as per SLEDAI-2 K definition. ^c^Pyuria present as per SLEDAI-2 K definition. SLEDAI: Systemic Lupus Erythematosus Disease Activity Index-2000; eGFR: estimated glomerular filtration rate; anti-dsDNA: anti-double-stranded DNA.
